# Celecoxib increases lung cancer cell lysis by lymphokine-activated killer cells via upregulation of ICAM-1

**DOI:** 10.18632/oncotarget.5745

**Published:** 2015-10-22

**Authors:** Melina Schellhorn, Maria Haustein, Marcus Frank, Michael Linnebacher, Burkhard Hinz

**Affiliations:** ^1^ Institute of Toxicology and Pharmacology, Rostock University Medical Center, Rostock, Germany; ^2^ Electron Microscopy Center, Rostock University Medical Center, Rostock, Germany; ^3^ Section of Molecular Oncology and Immunotherapy, Department of General Surgery, Rostock University Medical Center, Rostock, Germany

**Keywords:** celecoxib, intercellular adhesion molecule 1, lung cancer cells, immune surveillance, lymphokine-activated killer cells

## Abstract

The antitumorigenic mechanism of the selective cyclooxygenase-2 (COX-2) inhibitor celecoxib is still a matter of debate. Using lung cancer cell lines (A549, H460) and metastatic cells derived from a lung cancer patient, the present study investigates the impact of celecoxib on the expression of intercellular adhesion molecule 1 (ICAM-1) and cancer cell lysis by lymphokine-activated killer (LAK) cells. Celecoxib, but not other structurally related selective COX-2 inhibitors (i.e., etoricoxib, rofecoxib, valdecoxib), was found to cause a substantial upregulation of ICAM-1 protein levels. Likewise, ICAM-1 mRNA expression was increased by celecoxib. Celecoxib enhanced the susceptibility of cancer cells to be lysed by LAK cells with the respective effect being reversed by a neutralizing ICAM-1 antibody. In addition, enhanced killing of celecoxib-treated cancer cells was reversed by preincubation of LAK cells with an antibody to lymphocyte function associated antigen 1 (LFA-1), suggesting intercellular ICAM-1/LFA-1 crosslink as crucial event within this process. Finally, celecoxib elicited no significant increase of LAK cell-mediated lysis of non-tumor bronchial epithelial cells, BEAS-2B, associated with a far less ICAM-1 induction as compared to cancer cells. Altogether, our data demonstrate celecoxib-induced upregulation of ICAM-1 on lung cancer cells to be responsible for intercellular ICAM-1/LFA-1 crosslink that confers increased cancer cell lysis by LAK cells. These findings provide proof for a novel antitumorigenic mechanism of celecoxib.

## INTRODUCTION

Celecoxib is a selective inhibitor of the prostaglandin (PG)-synthesizing enzyme cyclooxygenase-2 (COX-2) [1]. Owing to its analgesic and anti-inflammatory effects, the selective COX-2 inhibitor (coxib) was approved for the symptomatic treatment of pain associated with rheumatoid arthritis and arthrosis in 1998. In addition, a 6-months treatment with a daily dose of 800 mg celecoxib was demonstrated to result in significant reductions of colorectal polyps in patients with familial adenomatous polyposis (FAP) [2], resulting in celecoxib's approval for adjuvant treatment of FAP patients by the US Food and Drug Administration in 1999. In matter of lung cancer reports have suggested celecoxib as treatment and preventive option [3–6] and to enhance the response to preoperative paclitaxel and carboplatin in early-stage non-small cell lung cancer (NSCLC) [7]. Taken into account that lung cancer is worldwide the most common cancer in terms of both incidence and mortality and that the response and remission rates in NSCLC patients still remain relatively low [8], these findings may offer new pharmacotherapeutical options in this field.

On the cellular level celecoxib exerts its anticarcinogenic action primarily via induction of cancer cell apoptosis or inhibition of proliferation (for review see [9]). Increasing evidence suggests a significant part of this action to occur independent of celecoxib's COX-2 inhibitory activity [9–13]. In a recent study, celecoxib was shown to even enhance COX-2 expression and PG formation by lung cancer cells as key events within its proapoptotic action [14]. However, the impact of celecoxib on cancer cell lysis resulting from tumor-immune interactions has been poorly investigated. In one study, celecoxib was found to produce a downregulation of major histocompatibility complex I molecule expression on metastatic breast cancer cells, thereby leading to improved recognition by natural killer (NK) cells conferring an enhanced tumor cell lysis [15].

The intercellular adhesion molecule 1 (ICAM-1), a glycoprotein consisting of five extracellular immunoglobulin-like domains, a transmembrane and a C-terminal intracellular domain [16], plays an important role in tumor immune surveillance and elimination of neoplastic cells [17]. There are several studies indicating cytokine-induced upregulation of ICAM-1 on cancer cells [18–23] or cancer cell transfection with the ICAM-1 gene [24, 25] to confer increased cytotoxic tumor cell lysis by immune cells. In the same context, a recent *in vitro* study suggests ICAM-1 upregulation as part of pharmacotherapeutic strategies. Accordingly, cannabinoids, a group of substances with diverse anticarcinogenic properties, have been shown to enhance the susceptibility of lung cancer cells to cytolytic death mediated by lymphokine-activated killer (LAK) cells via increase of ICAM-1 on cancer cell surface [26]. In line with its antitumorigenic responses observed *in vitro*, ICAM-1 expression has been likewise reported to be negatively correlated with metastasis of several cancer types in clinical studies [27–29].

The present study investigates the impact of celecoxib on tumor immune surveillance and the role of ICAM-1 within this process. Here we show that celecoxib, but not other structurally related COX-2 inhibitors, induces an upregulation of ICAM-1 expression on lung cancer cells, thereby causing increased cancer cell lysis by LAK cells. These findings provide evidence for a hitherto unknown mechanism underlying the anticarcinogenic action of celecoxib.

## RESULTS

### Celecoxib induces ICAM-1 expression on both protein and mRNA level

**Figure 1:**
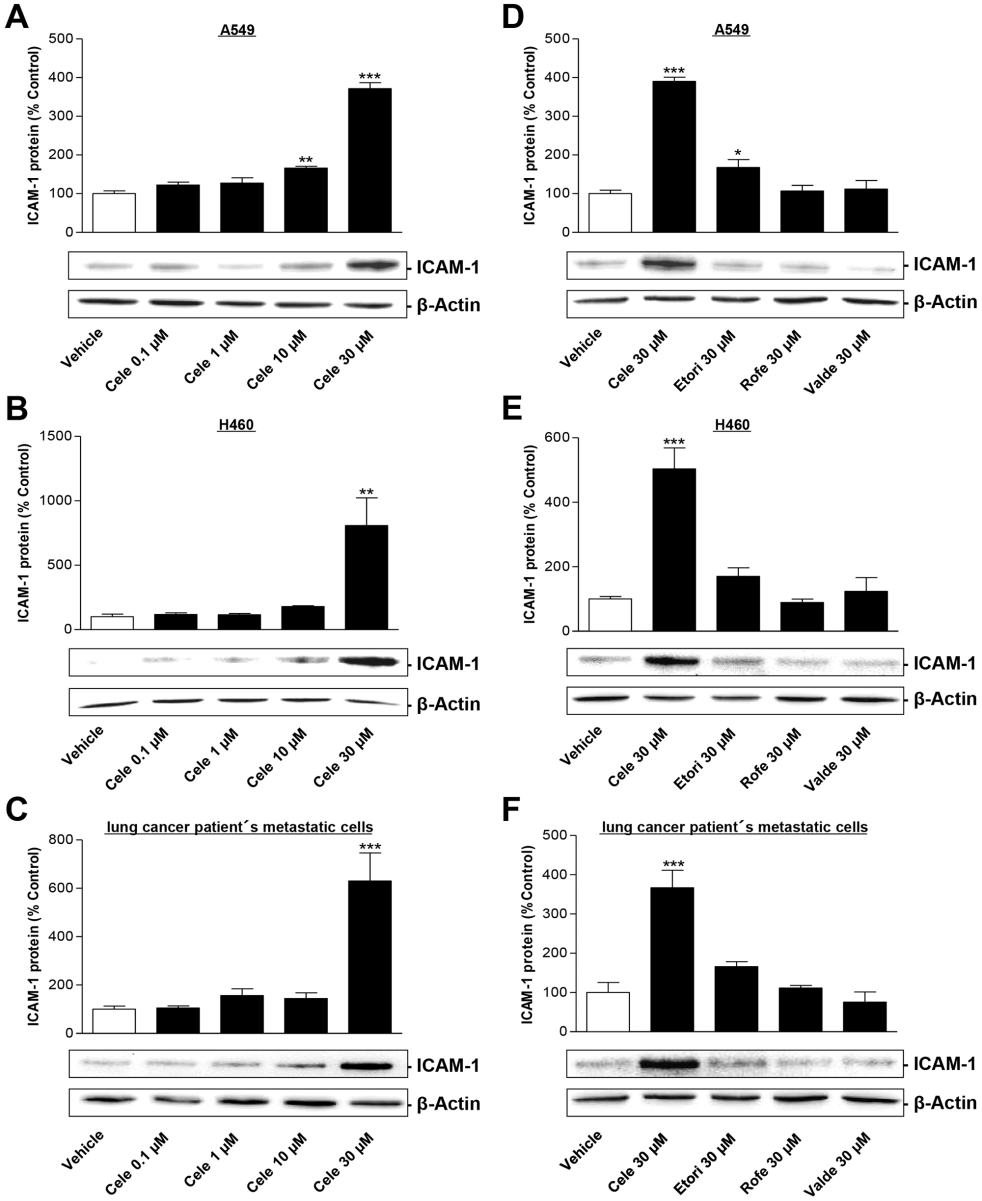
Effect of celecoxib and other selective COX-2 inhibitors on ICAM-1 protein expression in human lung cancer cells Left panels: Concentration-dependent impact of celecoxib on ICAM-1 protein expression in A549 **A.** H460 **B.** and lung cancer patient's metastatic cells **C.** Cells were incubated with celecoxib at the indicated concentrations for 48 h. Histograms above selected blots represent means ± SEM obtained from densitometric analysis of *n* = 4 (A, C) or *n* = 3 (B) blots. Right panels: Influence of selective COX-2 inhibitors on ICAM-1 protein expression in A549 **D.** H460 **E.** and lung cancer patient's metastatic cells **F.** Tumor cells were treated with 30 μM celecoxib (Cele), etoricoxib (Etori), rofecoxib (Rofe), valdecoxib (Valde) or vehicle for 48 h. Histograms above selected blots represent means ± SEM obtained from densitometric analysis of *n* = 4 (D, E, F) blots. *P < 0.05, **P < 0.01, ***P < 0.001; one-way ANOVA plus post hoc Dunnett test.

To investigate the impact of celecoxib on ICAM-1 expression and tumor cell lysis two human NSCLC cell lines (A549, H460) as well as metastatic cells derived from a lung cancer patient were used. In each of these cell types celecoxib was found to stimulate the protein expression of ICAM-1 (Fig. 1A–1C). According to an all-or-none principle this effect was significant after a treatment with 30 μM celecoxib in all three cell lines.

Additional experiments were performed to investigate the impact of three other structurally similar selective COX-2 inhibitors (etoricoxib, rofecoxib, valdecoxib) on ICAM-1 protein expression (Fig. 1D–1F). In fact, an upregulation of ICAM-1 protein greater than 3-fold was unique for celecoxib and was not shared by other selective COX-2 inhibitors. These findings are consistent with recently published data by our group indicating an upregulation of COX-2 expression by celecoxib, but not by other COX-2 inhibitors [14].

**Figure 2:**
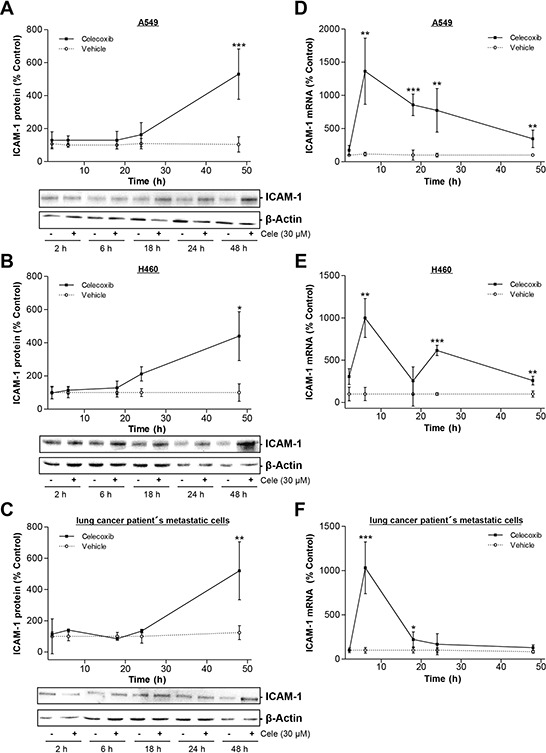
Time-dependent impact of celecoxib on ICAM-1 expression in A549, H460 and lung cancer patient's metastatic cells **A–C.** Western blot analysis of celecoxib's (30 μM) effect on ICAM-1 protein expression over a 48-h incubation period. Values are means ± SEM obtained from densitometric analysis of *n* = 3 blots. *P < 0.05, **P < 0.01, ***P < 0.001 vs. corresponding vehicle control of the respective ICAM-1 analysis; Student's *t* test. **D–F.** Real-time RT-PCR analysis of the impact of 30 μM celecoxib on ICAM-1 mRNA expression over a 48-h incubation period. Values are means ± SEM of *n* = 4 experiments. *P < 0.05, **P < 0.01, ***P < 0.001 vs. corresponding vehicle control of the respective ICAM-1 analysis; Student's *t* test.

Time-course experiments revealed a significant upregulation of ICAM-1 protein expression in lung cancer cells after a 48-h incubation with 30 μM celecoxib (Fig. 2A–2C). In accordance to elevated protein levels an increase of ICAM-1 mRNA level was detected after 6 h in each cell line (Fig. 2D–2F).

### Celecoxib increases LAK cell-mediated tumor cell lysis

To investigate the functional consequence of increased ICAM-1 expression by celecoxib, LAK cell-mediated tumor cell killing was investigated using a co-culture of LAK cells and pretreated cancer cells at a defined effector:target cell ratio (see Materials and Methods). Noteworthy, lymphocyte function associated antigen 1 (LFA-1), the cognate ICAM-1 receptor on the surface of immune cells, has recently been demonstrated to confer LAK cell-mediated killing of lung cancer cells incubated with the ICAM-1-upregulating phytocannabinoid cannabidiol before [26].

**Figure 3:**
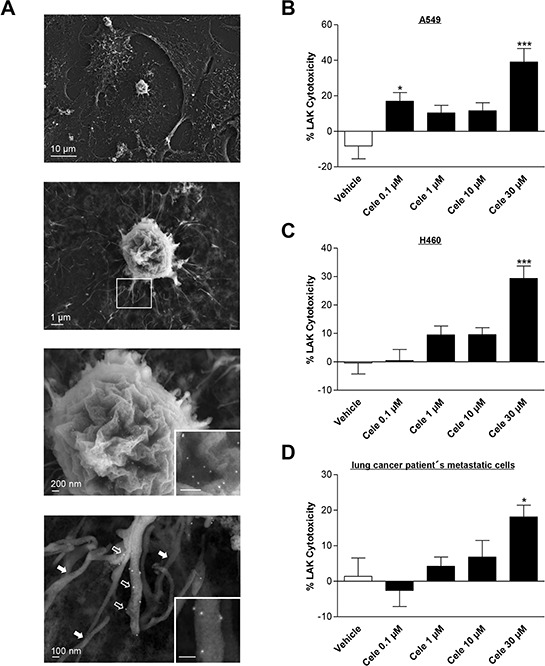
Effect of celecoxib on LAK cell-mediated tumor cell killing A series of scanning electron micrographs (**A.** left panels) visualizes the interactions between LAK cells and tumor cells. Electron micrographs at lower magnification show that LAK cells firmly attach to the spread A549 tumor cells with their processes (upper two panels). In addition, immunolabelling with LFA-1 antibody and a secondary antibody coupled to 15 nm colloidal gold was used to mark LAK cells. The gold labelling is visible as bright dots in the electron micrographs at higher magnifications (lower two panels with inserts) and decorates the cell body (second to last panel) as well as the processes of the LAK cell (lowermost panel, corresponding to the boxed area in the low magnification second from above panel). Note that the 15 nm gold labeling is confined to the processes of the LAK cell (open arrows) but is absent from the intermingled filopodia of the underlying tumor cell (filled arrows). Right panels: Concentration-dependent impact of celecoxib on LAK cell-mediated killing of A549 **B.** H460 **C.** or lung cancer patient's metastatic cells **D.** Tumor cells were incubated with celecoxib at the indicated concentrations for 48 h. Subsequently, these cells were co-incubated with LAK cells for 6 h. Values are means ± SEM of *n* = 24 (B, 6 donors), *n* = 28 (C, 7 donors) or *n* = 20 (D, 5 donors) experiments. *P < 0.05, ***P < 0.001 vs. corresponding vehicle control; one-way ANOVA plus post hoc Dunnett test.

The close interactions between tumor cells and LAK cells were visualized by scanning electron microscopy showing a firm attachment of the LAK cell with their processes to the tumor cell surface (Fig. 3A, upper two panels). The identity of LAK cells was verified by immuno-labelling using an LFA-1 antibody in conjunction with a secondary antibody coupled to 15 nm colloidal gold, detectable as bright dots by high resolution electron microscopy (Fig. 3A, lower two panels with inserts). The scanning electron microscopy analysis shows that gold grains indicating LFA-1 expression decorate the cell surface and processes of LAK cells (e.g., lowermost panel, open arrows), whereas the cell bodies and filopodial extensions of the underlying tumor cells are devoid of LFA-1 labelling (lowermost panel, filled arrows).

To address the impact of celecoxib on LAK cell-mediated tumor cell lysis, tumor cells that were incubated with increasing concentrations of celecoxib for 48 h were subsequently labeled with calcein-AM and co-cultured with LAK cells. Following a 6-h incubation, tumor cell lysis was measured by detection of calcein fluorescence in the supernatant. As shown in Fig. 3B–3D, celecoxib at 30 μM increased LAK cell-mediated tumor cell lysis of each tested lung cancer cell line. In some cases lysis of cancer cells appeared to be higher in the absence of LAK cells resulting in negative values of calculated percental LAK cytotoxicities, which is in line with observations from other groups [23, 30].

Recently, our group reported a toxic effect of celecoxib on lung tumor cells [14]. Thus, viability tests were performed. As compared to vehicle (100% viability), incubation of tumor cells with 30 μM celecoxib yielded viability rates of 64% ± 8% for A549, 49% ± 3% for H460 and 45% ± 4% for lung cancer patient's metastatic cells (all values as means ± SEM of *n* = 20–25 experiments).

### Celecoxib does not interfere with LAK cell function

**Figure 4:**
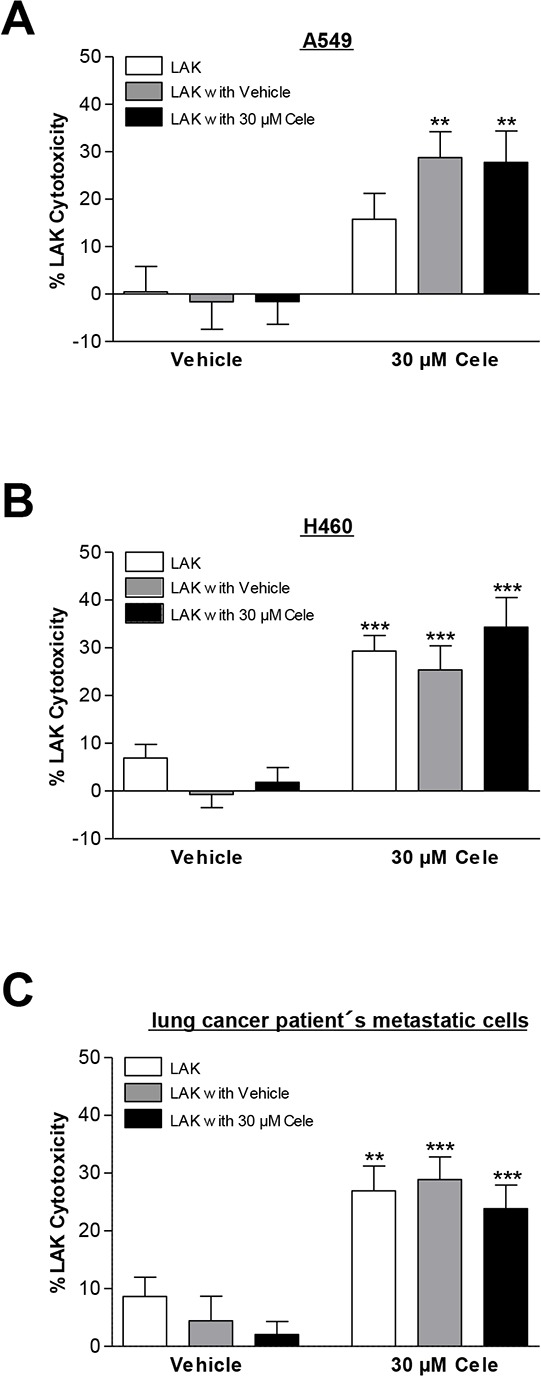
Impact of celecoxib on cytotoxic activity of LAK cells The respective subgroups indicate lysis of cancer cells when LAK cells were preincubated for 48 h in medium (white bars), medium containing vehicle (grey bars) or 30 μM celecoxib (black bars) prior to co-culturing with cancer cells. As LAK cell medium RPMI 1640 supplemented with 10% heat-inactivated FCS, 100 U/ml penicillin, 100 μg/ml streptomycin and 10 ng/ml IL-2 was used. Cytotoxicity was analyzed following a subsequent 6-h co-incubation of LAK cells with cancer cells. Values are means ± SEM of *n* = 12 (**A.** 3 donors), *n* = 20 (**B.** 5 donors) or *n* = 16 (**C.** 4 donors) experiments. **P < 0.01, ***P < 0.001 vs. corresponding vehicle control; Student's *t* test.

Further experiments were performed to address the impact of celecoxib on tumor cell lysis under conditions where LAK cells are exposed to this compound as would be the case under *in vivo* conditions. To this end, LAK cells prepared and cultured using the same protocol were additionally incubated with celecoxib or vehicle control for 48 h. However, the results obtained from these experiments (Fig. 4A–4C) revealed no influence of a pretreatment of LAK cells with celecoxib on LAK cell-mediated tumor cell lysis, implying the cytotoxic activity of LAK cells to be unaltered by celecoxib.

### ICAM-1 antibody suppresses celecoxib-induced LAK cell-mediated tumor cell lysis

**Figure 5:**
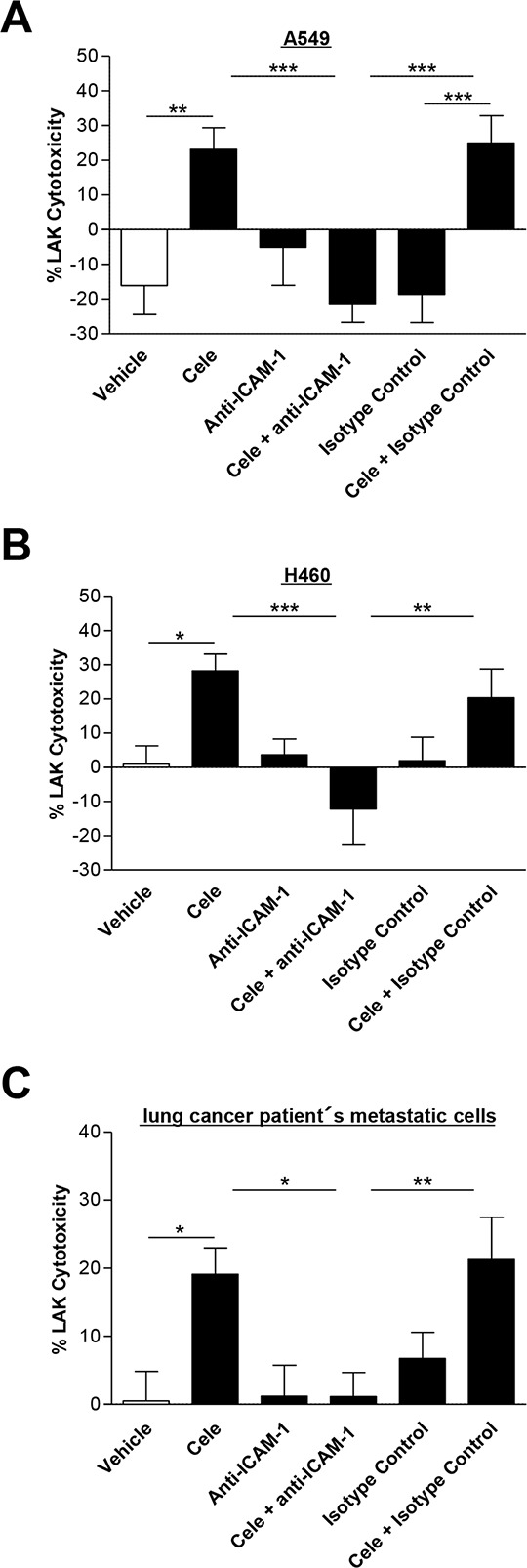
Effect of a neutralizing ICAM-1 antibody on cytotoxic lysis of cancer cells by LAK cells A549 **A.** H460 **B.** and lung cancer patient's metastatic cells **C.** were incubated with vehicle or 30 μM celecoxib for 48 h. Before starting cytotoxicity assay cancer cells were pre-incubated with an ICAM-1 antibody (1 μg/ml) for 2 h. An isotype control antibody (1 μg/ml) was used as negative control. Values are means ± SEM of *n* = 24 (A, 6 donors), *n* = 16 (B, 4 donors) or *n* = 20 (C, 5 donors) experiments. *P < 0.05, **P < 0.01, ***P < 0.001; one-way ANOVA plus post hoc Bonferroni test.

To confirm a causal link between celecoxib-induced upregulation of ICAM-1 protein expression and the concomitant increase of LAK cell-mediated tumor cell lysis by celecoxib, a neutralizing antibody to ICAM-1 was tested for its inhibitory action on tumor cell lysis. In all tumor cells investigated the ICAM-1 antibody significantly suppressed the celecoxib-induced tumor cell lysis by LAK cells when compared to cells treated with vehicle and isotype control antibody, respectively (Fig. 5A–5C). Noteworthy, both neutralizing ICAM-1 antibody and isotype control antibody did not alter the celecoxib-induced loss of cancer cell viability as assessed by WST-1 analysis (data not shown).

### LFA-1 antibody reverses celecoxib-induced tumor cell killing by LAK cells

**Figure 6:**
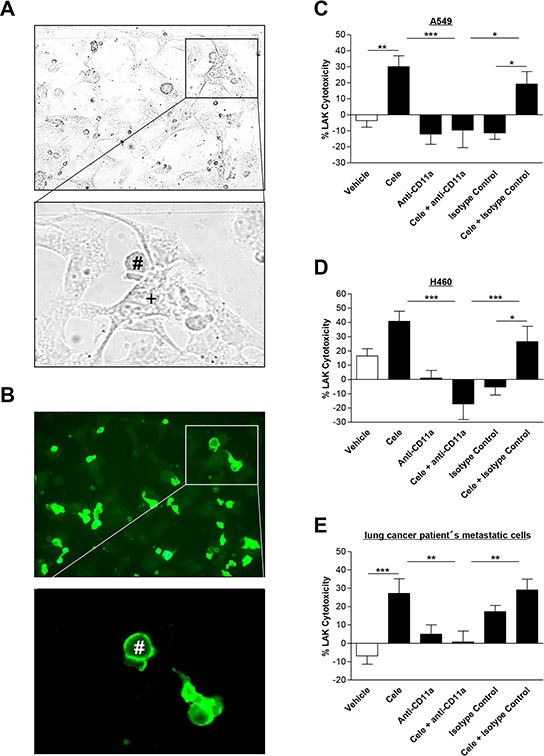
Effect of a CD11a (LFA-1) neutralizing antibody on interaction of cancer cells with LAK cells Light microscopy picture **A.** presents interaction between tumor (+) and LAK cells (#), whereas fluorescence microscopy pictures show immunocytochemical staining of LFA-1 expression on the surface of the immune cells (**B.** green fluorescence). Right panels: Influence of CD11a (LFA-1) antibody on cytotoxic lysis of cancer cells by LAK cells. A549 **C.** H460 **D.** and lung cancer patient's metastatic cells **E.** were incubated with vehicle or 30 μM celecoxib for 48 h. LAK cells were pre-incubated with a CD11a antibody (1 μg/ml) or an isotype control antibody (1 μg/ml) as negative control for 2 h before cytotoxicity assay was started. Values are means ± SEM of *n* = 20 (C, D, 5 donors) or *n* = 28 (E, 7 donors) experiments. *P < 0.05, **P < 0.01, ***P < 0.001; one-way ANOVA plus post hoc Bonferroni test.

The interaction of tumor and LAK cells is shown by light microscopy in Fig. 6A. According to immunocytochemical analysis (Fig. 6B), CD11a (LFA-1) is present on the surface of LAK cells (Fig. 6B, green fluorescence), but not detectable on the surface of tumor cells.

To verify an intercellular ICAM-1/LFA-1 crosslink as crucial event within the process of LAK cell-mediated lysis of celecoxib-treated tumor cells, LAK cells were preincubated with a neutralizing LFA-1 antibody for 2 h before killing assay was started. According to the histograms presented in Fig. 6C–6E, the neutralizing LFA-1 antibody significantly inhibited the effect of LAK cell-mediated tumor cell lysis by celecoxib. These results indicate LFA-1 as a potential receptor for ICAM-1 conferring LAK cell-mediated tumor cell lysis.

### Celecoxib does not affect human bronchial epithelial cells

**Figure 7:**
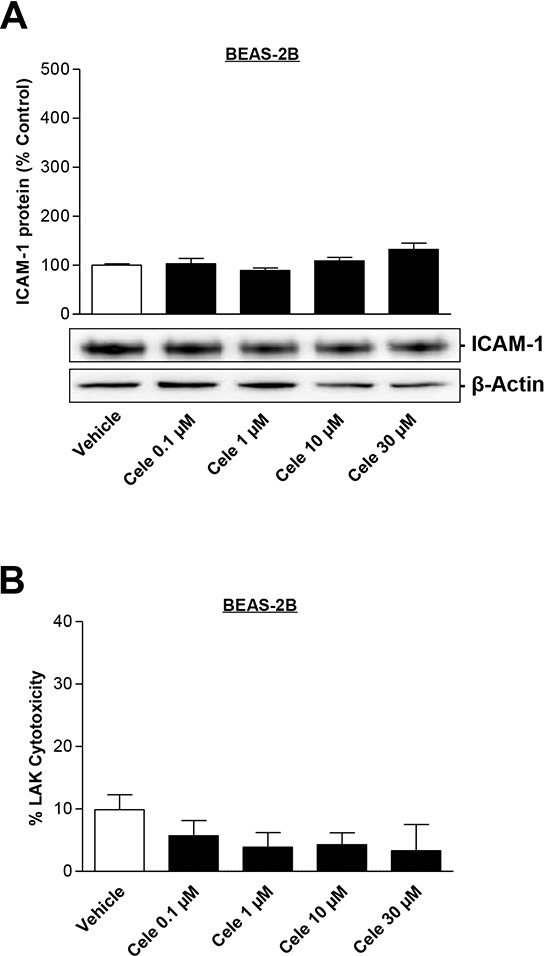
Concentration-dependent effect of celecoxib on ICAM-1 protein expression in human bronchial epithelial cells (BEAS-2B) and on cytotoxic lysis of BEAS-2B cells by LAK cells BEAS-2B cells were incubated with celecoxib at the indicated concentrations for 48 h. Histogram **A.** above the selected blot represents means ± SEM obtained from densitometric analysis of *n* = 5 blots. Values in **B.** are means ± SEM of *n* = 20 (5 donors) experiments. One-way ANOVA plus post hoc Dunnett test revealed no significant effect of celecoxib vs. vehicle.

To evaluate if celecoxib has any effect on non-tumor cells, the bronchial epithelial cell line BEAS-2B was used in further experiments. As shown in Fig. 7A, celecoxib did not cause a significant upregulation of ICAM-1 protein expression in BEAS-2B cells. In comparison, the same concentration of celecoxib tested under comparable experimental conditions resulted in an upregulation of ICAM-1 protein level in lung cancer cells (A549, H460, lung cancer patient's metastatic cells) by up to 3.7- (Fig. 1A), 8- (Fig. 1B) or 6.3-fold (Fig. 1C) over control. Additional experiments disproved an increase of LAK cell-mediated lysis of BEAS-2B cells treated with various concentrations of celecoxib for 48 h (Fig. 7B).

## DISCUSSION

The present study provides first-time proof for celecoxib to induce upregulation of the adhesion molecule ICAM-1 on the surface of tumor cells, resulting in increased tumor cell lysis by LAK cells.

There are several lines of evidence supporting this notion. First, celecoxib caused a substantial upregulation of ICAM-1 expression on both mRNA and protein level in lung tumor cell lines as well as in metastatic lung cancer cells. Second, celecoxib treatment of lung cancer cells resulted in an enhanced susceptibility to cytotoxic lysis by LAK cells. Third, the celecoxib-induced increase of LAK cell-mediated lysis of tumor cells was abrogated by neutralizing antibodies against ICAM-1 and LFA-1. The LFA-1 heterodimer (CD11a/CD18) is the natural ligand of ICAM-1 [31] that has been reported to represent an important link to conjugate ICAM-1-bearing cells with natural killer cells [32] and to confer lymphocyte-induced tumor cell killing [33]. The data presented here suggest LFA-1 as a crucial counter-receptor of ICAM-1 in conferring the celecoxib-induced enhanced susceptibility of lung cancer cells to LAK cell-mediated killing. Fourth, tumor cells were lysed by celecoxib-treated LAK cells to a comparable extent as by untreated or vehicle-treated LAK cells, thereby confirming celecoxib to enhance the susceptibility of tumor cells to LAK cell-induced killing while sparing direct effects on LAK cell activity. Overall, this data are in good line with several studies indicating enhanced ICAM-1 expression to confer an increased lysis of cancer cells by immune cells comprising NK and LAK cells, monocytes and tumor-infiltrating lymphocytes [18–26].

In the present study upregulation of ICAM-1 protein expression in lung cancer cells was confined to celecoxib and was not elicited by other related selective COX-2 inhibitors (i. e., etoricoxib, rofecoxib, valdecoxib) bearing a diaryl heterocyclic structure. This pattern is in agreement with a recent study from our group that shows a specificity of celecoxib among other selective COX-2 inhibitors in inducing lung cancer cell apoptosis and upregulating COX-2 expression [14]. Likewise, celecoxib, but not other selective COX-2 inhibitors, has been reported to induce apoptosis in synovial fibroblasts [34] and to cause antiproliferative effects on colon cancer cells and reduction of tumor growth *in vivo* [35].

Clearly, the concentrations of celecoxib causing ICAM-1 expression (i.e., 30 μM) or apoptosis in other studies (i.e., 40–100 μM) [9] exceed plasma concentrations of celecoxib, which have been reported to reach a maximum of 7.67 μM after single-dose administration of 800 mg celecoxib to human volunteers [1]. However, the unique effects of celecoxib within the coxibs may be due to an intracellular accumulation of this COX-2 inhibitor. Accordingly, celecoxib was detected at five- to ten-fold higher intracellular concentrations in different tumor cell types when compared to other coxibs (etoricoxib, lumiracoxib, rofecoxib, valdecoxib) [36]. According to Maier et al. [36] the intracellular accumulation of celecoxib results from integration into cellular phospholipid membranes and may thus provide a molecular basis for celecoxib's ability to interact with non-COX-2 targets *in vivo* despite comparatively low plasma concentrations [36]. For example, celecoxib at 50 μM has been reported to cause a COX-2-independent activation of the transcription factor nuclear factor κB [37, 38] that plays a pivotal role in ICAM-1 expression [39]. In addition, higher intracellular concentrations may be achieved *in vivo* through longer exposure times. Accordingly, cancer patients receive repeated treatment over weeks or months resulting in cumulative effects of the respective chemotherapy or radiation therapy [9, 40].

Upregulation of ICAM-1 is likewise supposed to mediate several adverse effects such as perpetuation of bronchial injury by adhesion of neutrophils to epithelial cells [41]. Consequently, the impact of celecoxib on healthy tissue was evaluated by use of the bronchial epithelial cell line BEAS-2B, which was established from normal bronchial epithelium of non-cancerous individuals [42]. However, in contrast to lung cancer cells celecoxib neither significantly affected ICAM-1 protein expression nor susceptibility of these cells against LAK cell-induced cytotoxicity. In line with this finding celecoxib was previously reported to impair the growth of colorectal cancer *in vivo* without causing toxic effects on normal gut epithelium [43].

In matter of ICAM-1 expression some studies indicate an inhibition of protein expression by celecoxib. Thus, celecoxib was shown to cause inhibition of ICAM-1 expression in colon cancer cells and a decreased adhesion to fetal calf serum (FCS)-coated plastic wells with both events mediated via a COX-2-independent pathway [44]. However, the maximal inhibitory effect of celecoxib (10 μM) on ICAM-1 expression occurred after a 4-h incubation with a maximal decrease of about 45% and already declined after 6 h [44]. In another investigation the same group found a 4-h incubation of tumor necrosis factor α-stimulated human umbilical vein endothelial cells with celecoxib to decrease ICAM-1 and vascular cell adhesion molecule 1 expression with a maximal inhibition of about 60% and 50% at 10 μM celecoxib, respectively, followed by reduced adhesion of colon cancer cells to endothelial cells [45]. Further studies indicate that celecoxib treatment of mice with experimentally induced atherosclerosis [46] and autoimmune encephalomyelitis [47] or rats with colitis and lung injury [48, 49] decreases the expression of ICAM-1. The reasons for this apparent discrepancy remain to be identified but may be explained, in case of *in vitro* studies, by the different experimental settings and specificity of cell types, respectively.

Altogether, the results of this study argue for an antitumorigenic function of ICAM-1. This view is further corroborated by animal studies that determined a 2.6-fold greater tumor volume in ICAM-1-deficient mice than in wild-type mice 14 days after injection of melanoma cells [50] or a development of malignant tumors in LFA-1-deficient but not wild-type mice injected with cancer cells [51]. In athymic nude mice the non-psychoactive cannabidiol elicited an increase of ICAM-1 protein in A549 xenografts and an antimetastatic effect that was fully reversed by a neutralizing antibody against ICAM-1 [52]. In other murine models ICAM-1 overexpression on tumor cells was found to elicit a reduced tumor growth [25, 53]. Analyses of primary tumors from patients with breast cancer revealed a negative correlation of ICAM-1 expression to tumor size, lymph node metastasis and tumor infiltration as well as a better relapse-free and overall survival in patients with ICAM-1-positive tumors than in those with negative tumors [27]. In line with this notion, the incidence of lymph node or liver metastasis was significantly lower in patients with ICAM-1-positive colorectal tumors than in those with ICAM-1-negative tumors [29]. Infiltration of tumor infiltrating lymphocytes was more frequently observed in the ICAM-1-positive tumors in this study [29]. In patients with gastric cancer ICAM-1 expression on cancer cells was significantly decreased in patients with lymph node metastasis with the prognosis of patients being poorer in patients with ICAM-1-negative tumors [28]. Other studies showed an association between ICAM-1 expression and infiltration of lymphocytes into tumor tissue of patients with renal cell carcinoma [54], colorectal [55] and esophageal cancer [56].

Collectively, the present study identified upregulation of ICAM-1 expression in lung cancer cells leading to LAK cell-mediated tumor cell lysis as a novel antitumorigenic mechanism of celecoxib. Further studies addressing the impact of celecoxib on tumor immune surveillance *in vivo* are suggested to better understand the pharmacological action of this drug.

## MATERIALS AND METHODS

### Materials

Celecoxib, etoricoxib, valdecoxib, aprotinin, calcein-AM, luminol, orthovanadate and phenylmethylsulfonyl fluoride (PMSF) were purchased from Sigma-Aldrich (Taufkirchen, Germany). Rofecoxib was obtained from Enzo Life Sciences (Lörrach, Germany). Leupeptin was bought from Biomol (Hamburg, Germany). Dimethyl sulfoxide (DMSO), ethylenediaminetetraacetic acid (EDTA), glycerol, hydrogen peroxide (H_2_O_2_), sodium chloride (NaCl), Tris hydrocloride (Tris-HCl), Tris ultrapure, calcium chloride dihydrate and magnesium chloride hexahydrate were obtained from AppliChem (Darmstadt, Germany). Dulbecco's modified Eagle's medium (DMEM) with 4.5 g/l glucose and with 4 mM L-glutamine and Roswell Park Memorial Institute medium (RPMI 1640) with L-glutamine were provided by Lonza (Cologne, Germany). FCS and penicillin-streptomycin were purchased from Invitrogen (Darmstadt, Germany) and phosphate-buffered saline (PBS) was provided by PAN Biotech (Aidenbach, Germany). Lymphocyte Separation Medium LSM 1077 was obtained from PromoCell (Heidelberg, Germany) and recombinant human interleukin-2 (IL-2) was supplied by ReliaTech (Wolfenbüttel, Germany). Triton^®^ X-100 and paraformaldehyde were bought from Roth (Karlsruhe, Germany). Neutralizing ICAM-1/CD54 antibody and isotype control antibody were purchased from R&D Systems (Wiesbaden-Nordenstadt, Germany). LEAF™ Purified anti-human CD11a and LEAF™ Purified Mouse IgG1, κ Isotype Control were supplied by BioLegend (London, UK). Alexa Fluor^®^ 488 conjugated F(ab′)2-Goat anti-Mouse IgG (H+L) Secondary Antibody was obtained from Life Technologies (Darmstadt, Germany). Normal Goat Serum (NGS) (Nr. B11–035) and glutaraldehyde were bought from PAA Laboratories (Pasching, Austria) and Electron Microscopy Sciences (Hatfield PA, USA), respectively. Goat anti-Mouse IgG (H&L) 15 nm gold antibody (Nr. EMGMHL 15) and fish gelatin (Nr. GEL 10) were provided by BBI Solutions (Cardiff, UK).

### Cell culture

The NSCLC cell lines A549 and H460, the lung cancer patient's metastatic cells as well as the human bronchial epithelial cell line BEAS-2B (ATCC-LGC, Wesel, Germany) were maintained in DMEM supplemented with 10% heat-inactivated FCS, 100 U/ml penicillin and 100 μg/ml streptomycin. A549 human lung carcinoma cells were purchased from DSMZ (Braunschweig, Germany; A549: DSMZ no.: ACC 107, species confirmation as human with IEF of MDH, NP; fingerprint: multiplex PCR of minisatellite markers revealed a unique DNA profile). H460 cells were purchased from ATCC-LGC (Wesel, Germany; ATCC™ Number: HTB-177™; cell line confirmation by cytogenetic analysis). Following resuscitation of frozen cultures none of the cell lines was cultured longer than 6 months.

Lung cancer patient's metastatic cells were obtained from resection of brain metastasis of a 47-year-old female Caucasian with NSCLC with the procedure of cell preparation described recently [52]. The patient had been informed about the establishment of cellular models from its tumor and had given informed consent in written form. The procedure was approved by the institutional ethical committee. Experiments were performed using passages 2–15 of these cells.

Cells were grown in a humidified incubator at 37°C and 5% CO_2_. All incubations with test substances were performed in serum-free medium. PBS was used as vehicle for COX-2 inhibitors with a final concentration of 0.1% (v/v) DMSO. As vehicle control PBS containing 0.1% (v/v) DMSO was used. The neutralizing ICAM-1/CD54 antibody and the isotype control antibody were dissolved in PBS. The LEAF™ Purified anti-human CD11a and LEAF™ Purified Mouse IgG1, κ Isotype Control antibody were supplied soluted in 0.2 μm filtered PBS (pH 7.2), containing no preservative and an endotoxin level < 0.01 ng/μg of the protein. For further dilutions PBS was used. For all antibody approaches PBS was used as vehicle control.

### Generation of LAK cells

Peripheral blood mononuclear cells (PBMCs) were isolated from buffy coats of healthy donors. A volume of 50–70 ml of each buffy coat was diluted 1:2 with PBS, carefully poured over 20 ml of Lymphocyte Separation Medium (LSM 1077) and centrifuged at 1171 × g for 25 min; no brake was applied during deceleration. Following centrifugation lymphocytes concentrating in the interphase (white phase) were collected and washed twice with PBS. Washing was performed by centrifugation at 300 × g for 10 min in the first step and 200 × g for 10 min in the second step. After centrifugation pellets were resuspended in RPMI 1640 supplemented with 10% heat-inactivated FCS, 100 U/ml penicillin and 100 mg/ml streptomycin. Adherent cells were removed from PBMC suspensions (2 × 10^6^ cells/ml) by attachment to plastic at the flask bottom for 1–2 h. This procedure was repeated once more before cells in the culture supernatant were subjected to further treatment. For generation of LAK cells the cell suspension was incubated with 10 ng/ml IL-2 for 6 days at a density of about 1.5 × 10^6^ cells/ml. After 3 days the medium was changed and fresh IL-2 was added.

For some experiments fractions of LAK cells were treated with vehicle or celecoxib. In this case vehicle or celecoxib was added to LAK cell suspension into the culture flask 48 h before starting the LAK cell cytotoxicity assay.

### LAK cell cytotoxicity assay

The cytotoxicity of LAK cells on tumor or BEAS-2B cells was determined by the calcein-AM release assay. Tumor or BEAS-2B cells (target cells) were seeded into 96-well flat bottom plates at a density of 1 × 10^4^ cells/well and were allowed to grow for 24 h. Cells were washed with PBS and treated with vehicle or test substance in serum-free DMEM. Following a 48-h incubation period, target cells were washed and labeled with 5 μM calcein-AM for 30 min. Subsequently, cells were washed with PBS and generated LAK cells (effector cells) were added to target cells at an effector:target cell ratio of 4:1 in a final volume of 100 μl/well. After a 6-h incubation (37°C, 5% CO_2_) supernatants were transferred to other wells of the 96-well plate and remaining target cells were lysed with 2% (v/v) Triton^®^ X-100. The fluorescence of supernatants and lysed target cells was recorded using a 485 nm excitation filter and a 535 nm emission filter by a Tecan infinite pro200 plate reader. LAK cell-induced cytotoxicity was monitored by the release of calcein by cancer cells into the supernatant due to toxic effects induced by LAK cells in the co-culture. To account for a probable modulation of cancer cell viability by vehicle or test substances, the fluorescence of cancer cells in the absence of effector cells, referred to as spontaneous release of calcein, was subtracted from these values. Finally, values were normalised to the release of calcein that can be achieved maximally by the effector cells. The percentage of LAK cytotoxicity was calculated as follows: % LAK cytotoxicity = (fluorescence of supernatant of sample well with effector cells - fluorescence of spontaneous release) / (fluorescence of maximal release - fluorescence of spontaneous release) [18]. Blank values were subtracted from the experimental data. Before LAK cytotoxicity was calculated the raw data of the fluorescent measurement were analysed with Nalimov test and outliers were excluded. In parallel to the LAK cell cytotoxicity assay viability of tumor cells was determined under equal conditions using the WST-1 assay (Roche, Grenzach-Wyhlen, Germany).

Experiments to determine the functional involvement of ICAM-1 in enhanced LAK cell-mediated tumor cell killing were performed using a neutralizing antibody against ICAM-1. Experiments with the ICAM-1 neutralizing antibody were performed by incubation of target cells with 1 μg/ml of an ICAM-1 antibody or an isotype control antibody as negative control for 2 h. Following preincubation of cancer cells with the antibodies, supernatants were removed carefully and without washing the co-incubation with LAK cells was started. For analysis of the involvement of LFA-1 in LAK cell-mediated tumor cell lysis a CD11a antibody or an isotype control antibody was used. Experiments were carried out by incubation of LAK cells with 1 μg/ml of the respective antibody for 2 h before starting cytotoxicity assay by adding the LAK cell suspension with the therein containing antibody to the target cells.

### Quantitative RT-PCR analysis

Lung cancer cells were seeded into 24-well plates at a density of 1 × 10^5^ cells/well and allowed to grow for 24 h. Following incubation of cells with celecoxib or its vehicle for the indicated times, cell culture media were removed and cells were lysed for subsequent RNA isolation. Total RNA was isolated using the RNeasy total RNA Kit (Qiagen, Hilden, Germany). β-Actin- (internal standard) and ICAM-1 mRNA levels were determined by quantitative real-time RT-PCR using the TaqMan^®^ RNA-to-C_T_™ Kit (Applied Biosystems, Darmstadt, Germany) according to the manufacturer's instruction. Primers and probe for human β-actin and ICAM-1 were Gene Expression Assay™ products (Applied Biosystems, Darmstadt, Germany).

### Western blot analysis

To analyze protein levels of ICAM-1, lung tumor or non-tumor cells were grown in 6-well plates at a density of 2 × 10^5^ cells/well for 24 h and subsequently incubated with vehicle or test substance for 48 h. After incubation cells were washed with PBS, harvested and lysed in solubilization buffer (50 mM HEPES, 150 mM NaCl, 1 mM EDTA, 1% (v/v) Triton^®^ X-100, 10% (v/v) glycerol, 1 mM PMSF, 1 mM orthovanadate, 1 mg/ml leupeptin, 10 mg/ml aprotinin). Lysis was performed for at least 30 min on ice and frequently mixing on a vortex mixer. Subsequently, lysates were centrifuged at 10,000 × g for 5 min and supernatants were then used for Western blot analysis. Total protein of cell lysates was determined using the bicinchoninic acid assay (Pierce, Rockford, USA). Proteins were separated using 10% sodium dodecyl sulfate (SDS) polyacrylamide gels and then transferred to nitrocellulose membranes (Roth, Karlsruhe, Germany) that were blocked with 5% milk powder (BioRad, Munich, Germany). Membranes were incubated with a primary mouse monoclonal antibody raised to ICAM-1 (Santa Cruz Biotechnology, Heidelberg, Germany) at 4°C overnight. Subsequently, blots were probed with horseradish peroxidase-conjugated anti-mouse IgG (New England Biolabs GmbH, Frankfurt am Main, Germany) and incubated for 1 h at room temperature. Antibody binding was visualized by a chemiluminiferous solution (100 mM Tris-HCl pH 8.5, 1.25 mM luminol, 200 mM p-coumaric acid, 0.09% [v/v] H_2_O_2_, 0.0072% [v/v] DMSO). Densitometric analysis of band intensities was achieved by optical scanning and quantifying using the Quantity One 1-D Analysis Software (Bio-Rad, Munich, Germany). To identify the band size of the Western blots, the prestained SDS-PAGE Standard (Broad Range; Bio-Rad, Munich, Germany) was used. A regression of the prestained standard revealed a band size of ICAM-1 at 90 kDa and of β-actin at 42 kDa. Vehicle controls were defined as 100% for evaluation of changes in protein expression. To ascertain equal protein loading in Western blots of cell lysates, membranes were probed with an antibody raised to β-actin (Sigma-Aldrich). All densitometric values were normalized to β-actin.

### Analysis of CD11a with fluorescence microscopy

For imaging of CD11a A549 cells were seeded at a density of 1–1.5 × 10^5^ cells/ml in 4-well culture slides (BD Falcon™, Heidelberg, Germany). After 3 days tumor cells were incubated with LAK cells at an effector:target cell ratio of 4:1 for 3 h. Subsequently, cells were fixed with 4% paraformaldehyde overnight, washed with PBS and blocked with PBS containing 0.3% (v/v) Triton^®^ X-100 and 5% (v/v) FCS for 1 h. After washing with PBS, cells were incubated with a CD11a antibody (1:250) for 1 h. For this purpose the same antibody as in the LAK cell cytotoxicity assay was used. As secondary antibody a goat anti-mouse Alexa Fluor^®^ 488-labeled IgG (1:1000) was used and incubation took place for 1 h as well. All antibodies were diluted in PBS containing 0.3% (v/v) Triton^®^ X-100 and 1% (v/v) FCS. Notably, experiments with secondary antibody were performed in the dark.

### Electron microscopy

The NSCLC cell line A549 was seeded on Melinex^®^ films (Plano, Wetzlar, Germany) at a density of 5 × 10^5^ cells per well in a 24-well plate. After tumor cells were co-cultured with LAK cells (effector:target cell ratio of 4:1) for 3 h, cells were carefully washed with PBS containing magnesium and calcium (each at 1 mM). Afterwards, cells were fixed with 4% paraformaldehyde containing magnesium and calcium (each at 1 mM) overnight. Subsequently, cells were blocked with 5% (v/v) NGS in PBS for 30 min and incubated with the primary CD11a (LFA-1) antibody (1:250) diluted in PBS containing 5% (v/v) NGS. For this purpose the same antibody as in the LAK cell cytotoxicity assay was used. After 1 h of incubation cells were washed with PBS containing 0.1% (v/v) Tween^®^ 20 before secondary goat anti-mouse antibody 15 nm gold (1:50) diluted in PBS containing 5% (v/v) NGS, 0.1% (v/v) Tween^®^ 20 and 0.1% (v/v) fish skin gelatin was added for 1 h. Cells were washed with PBS containing 5% (v/v) NGS, 0.1% (v/v) Tween^®^ 20 and 0.1% (v/v) fish skin gelatin and post-fixed with 2.5% (v/v) glutaraldehyde.

For scanning electron microscopy, the film supports with the attached cells were washed with 0.1 M sodium phosphate buffer pH 7.3 and were subsequently dehydrated in a graded series of acetone. Critical point drying was performed in an Emitech K850 critical point dryer (Emitech Ltd. Ashford, UK) after three rounds of immersion in CO_2_. The dried film supports were mounted on scanning electron microscopy stubs with adhesive carbon tape (Plano, Wetzlar, Germany) and coated with a carbon layer using a Leica SCD500 coater (Leica Microsystems, Wetzlar, Germany). Specimens were viewed in a Merlin VP compact scanning electron microscope (Carl Zeiss Microscopy, Jena, Germany) operated at 5 kV. Images with a size of 1024 × 768 and 2048 × 1536 pixels were recorded with the SmartSEM Software (Carl Zeiss Microscopy) and were processed with Adobe Photoshop CS6 (Adobe Inc. San Jose, CA, USA).

### Statistical analysis

Comparisons between groups were performed with Student's two-tailed *t* test or with one-way ANOVA plus post hoc Bonferroni or Dunnett test using GraphPad Prism 5.0 (GraphPad Software, Inc., San Diego, USA).

## Abbreviations

COX: cyclooxygenase
ICAM-1: intercellular adhesion molecule 1
IL-2: interleukin-2
LAK cells: lymphokine-activated killer cells
LFA-1: lymphocyte function associated antigen 1
NSCLC: non-small cell lung cancer
